# Study of LAT1 Expression in Brain Metastases: Towards a Better Understanding of the Results of Positron Emission Tomography Using Amino Acid Tracers

**DOI:** 10.1371/journal.pone.0157139

**Published:** 2016-06-08

**Authors:** Caroline Papin-Michault, Christelle Bonnetaud, Maxime Dufour, Fabien Almairac, Mickael Coutts, Stéphanie Patouraux, Thierry Virolle, Jacques Darcourt, Fanny Burel-Vandenbos

**Affiliations:** 1 Department of Pathology, University Hospital, Nice, France; 2 Department of Experimental Pathology, University Hospital, Nice, France; 3 Department of Nuclear Medicine, Centre Antoine Lacassagne, Nice, France; 4 TIRO–UMR E 4320, University of Medicine, Nice, France; 5 Department of Neurosurgery, University Hospital, Nice, France; 6 UMR CNRS 7277-UMR INSERM 1091, Institute of Biology Valrose, University of Sciences, Nice, France; Wake Forest University, School of Medicine, UNITED STATES

## Abstract

Positron emission tomography using radiolabeled amino acid (PET-AA) appears to be promising in distinguishing between recurrent tumour and radionecrosis in the follow-up of brain metastasis (BM). The amino acid transporter LAT1 and its cofactor CD98, which are involved in AA uptake, have never been investigated in BM. The aim of our study was to determine and compare the expression of LAT1 and CD98 in BM and in non-tumoral brain tissue (NT). The expression of LAT1 and CD98 were studied by immunohistochemistry in 67 BM, including 18 BM recurrences after radiotherapy, in 53 NT, and in 13 cases of patients with previously irradiated brain tumor and investigated by [18F] FDOPA-PET. LAT1 and CD98 expression were detected in 98.5% and 59.7% of BM respectively and were significantly associated with BM tissue as compared to NT (p<0.001). LAT1 expression in recurrent BM was significantly increased as compared to newly occurring BM. Ten cases investigated by [18F] FDOPA-PET corresponding to recurrent BM displayed significant [18F] FDOPA uptake and LAT1 overexpression whereas three cases corresponding to radionecrosis showed no or low uptake and LAT1 expression. LAT1 expression level and [18F] FDOPA uptake were significantly correlated. In conclusion, we hypothesized that BM may overexpress the AA transporter LAT1. We have shown that LAT1 overexpression was common in BM and was specific for BM as compared to healthy brain. These results could explain the specific BM uptake on PET-AA.

## Introduction

Brain metastases (BM) are a grave complication of systemic cancers and represent the commonest type of brain tumor. The most common tumors that metastasize to the brain are tumors of the lung (50–60% of brain metastases), the breast (15–20%), melanoma (5–20%), renal (5–10%), and colorectal (6%) tumors [1]. BM often occur at an advanced stage of the disease but may be the presenting lesion. The prognosis of cancers with BM is generally poor, with a median survival of several weeks in untreated patients but often improve to several months with oncological treatment. Radiotherapy plays an important role in the management of BM, either as whole-brain radiation therapy (WBRT) or as stereotactic radiosurgery (SRS) according to the number of BM and the performance status of the patient. Surgery, either alone or combined with radiotherapy, may be indicated for oligometastatic brain disease. Despite treatment, the recurrence of BM is very common. The diagnosis of recurrent BM may be challenging with pseudoprogressive features on imaging due to radionecrosis. Due to the fact that some recurrent BM may be re-treated by radiotherapy or surgery, it is crucial to distinguish BM from radionecrosis.

MRI is the gold standard for the evaluation of brain lesions but it is often insufficient to differentiate conclusively between tumor recurrence and radionecrosis. Overcoming this problem requires the use of other techniques providing metabolic data. Positon emission tomography (PET) has an increasing role in the diagnosis of cancer [2]. [18F] FDG- PET is the most frequently used PET modality for cancer staging. As a result of a physiological uptake of glucose by normal brain tissue, [18F] FDG-PET lacks specificity in the assessment of brain lesions. [18F] FDG-PET lacks sensitivity for the detection of tumors with low glucose metabolism such as low grade glioma because [18F] FDG uptake in low grade tumors is usually similar to that of normal white matter [3]. In the same way, some recurrent tumors after treatment may have a low glucose metabolism and may be difficult to diagnose using [18F] FDG-PET. Furthermore, as [18F] FDG uptake increases in inflammatory lesion, [18F] FDG-PET may not be sufficiently specific to differentiate an inflammatory lesion from a tumor. Diagnostic performance of [18F] FDG-PET has been reported to be poor for the differentiation between tumor recurrence and radionecrosis [4]. PET using amino acid or analog tracers (AA-PET), such as 11C-Methionine (MET), 18F-Fluorothymidine (FLT), 18F-Fluoro-L-Tyrosine (FET) or 18F-fluoro-dihydroxy-L-phenylalanine (F-DOPA), overcomes this problem of physiological brain uptake. There is an increasing interest in these techniques using AA tracers and AA-based radiotracers continue to be developed in order to improve imaging properties relative to [18F] FDG-PET [5,6]. They are attractive for brain imaging because of a high uptake of tumor tissues as compared to a low uptake of normal brain. Furthermore, AA uptake is increased in both low- and high grade tumors [3,4]. A number of studies suggested that AA-PET would be useful in the follow-up of gliomas, distinguishing recurrent glioma from radionecrosis [3,7]. Other studies have shown promising results with AA-PET in the distinction of recurrent BM and radionecrosis, with a sensitivity ranging from 78% to 95% and a specificity ranging from 75% to 100% [8–12]. Lizarraga *et al*.[12] showed, in a series of 32 patients, that [18F] FDOPA-PET was useful for differentiating recurrent BM from radionecrosis, with a sensitivity of 81.3% and a specificity of 84.3%. Recently, Cicone *et al*.[13] confirmed these findings in a series of 50 BM and found a sensitivity of 90% and a specificity of 92.3%. Conversely, there are no biological data explaining the specific AA uptake by BM observed in AA-PET as compared to non tumoral brain.

The L-type amino acid transporter 1 (LAT1) is a membrane 55kDa glycoprotein, involved in the cellular capture of large neutral or aromatic AA, such as Leu, Ile, Phe, Met, Tyr, His, Trp, Val, and amino acid-related compounds such as L-DOPA, which it transports with a high affinity [14,15]. LAT1 is involved in both uptake and efflux of these AA and functions as an amino-acid exchanger [15]. Its transport capacity is independent of sodium but is affected by extracellular pH, with activities increasing in low pH conditions [16,17]. In order to perform its role as an AA transporter, LAT1 needs the cofactor 4F2hc (or CD98), with which LAT1 forms a heterodimeric complex via a disulfide bond. This is the main mechanism of AA uptake in AA-PET [18–20]. Physiological LAT1 expression is restricted to the gonads, placenta and to the blood-brain barrier (BBB) [14,15,21]. The expression of LAT1 and of CD98 increases in numerous tumors and this correlates with hypermetabolism of tumor cells requiring increased absorption of AA for growth and proliferation [14,15,22]. Furthermore, their expression in some cancers is correlated with more aggressive tumor behavior, more advanced stage of disease and with a worse prognosis [23–28]. For these reasons, we hypothesized that LAT1 and CD98 could be overexpressed in BM. The principal aim of our study was to determine the frequency and the level of LAT1 and CD98 expression in BM from various origins. A secondary aim was to determine if LAT1 and CD98 expression were specific for tumoral tissue in comparison to non-tumoral brain.

## Materials and Methods

### Tissue and lesions

Retrieval of BM tissues and non-tumoral brain tissues (NT) was facilitated by the APIX database of the Department of Pathology, University Hospital of Nice. All specimens were excisions with histology performed. A total of 49 consecutively operated BM without preoperative radiotherapy were selected during the period between June 2011 and September 2012 together with 18 consecutive cases of post-radiotherapy recurrent BM during the period between November 2007 and September 2012. Among the 53 cases of NT, 37 cases corresponded to peri-metastatic gliosis and 16 cases corresponded to peri-abscess (14 cases) or peri-hematoma (1 case) gliosis and radionecrosis (1 case). BM and lesions are detailed in Table 1. All of the samples are the property of the tissue collection of the Pathology department, which is declared annually to the French Health Ministry. The procedures followed were approved by the institutional review board of the University Hospital of Nice.

**Table 1:** Description of tissues and lesions.

Brain metastases* (n = 67)		Non tumoral brain tissues (n = 53)	
Non recurrent BM* (n = 49)	Post-radiotherapy recurrent BM* (n = 18)	Non irradiated (n = 40)	Irradiated (n = 13)
22 lung adenocarcinomas	10 lung adenocarcinomas	25 peri-metastatic gliosis	12 irradiated peri-metastatic tissues
8 lung squamous carcinomas	4 lung squamous carcinomas	14 peri-abcess gliosis	1 radionecrosis
6 lung large cell carcinomas	3 lung large cell carcinomas	1 peri-hematoma gliosis	
2 lung small cell carcinomas	1 colonic adenocarcinoma		
4 colonic adenocarcinomas			
2 melanomas			
1 renal clear cell carcinoma			
1 breast adenocarcinoma			
1 prostatic adenocarcinoma			
2 carcinomas of unknown origin			

* Brain metastases

### Immunohistochemistry

LAT1 and CD98 immunohistochemistry was performed on paraffin-embedded tumor sections using a rabbit monoclonal antibody for LAT1 (clone EPR3492, OriGen Technologies, 1:250) and a rabbit polyclonal antibody for CD98 (Thermo Scientific, 1:100). Deparaffinization, rehydration and antigen retrieval were performed using the pretreatment module PTlink (Dako) at pH6 and pH9 respectively. Primary antibodies were incubated for 20 min. For CD98, signal amplification was performed using Dako EnVision^TM^ Flex+ Rabbit Linker. Revelation was performed using the revelation kit Dako EnVision^TM^ Flex/HRP, with diaminobenzidin as chromogen. Sections were counterstained with haematoxylin.

LAT1 and CD98 immunostaining were considered positive when at least 10% of cells displayed membrane staining. For each positive case, a score based on the Hirsch score [29] was determined ranging between 0 and 400. Microvessel staining was used both as a positive control and for the evaluation of staining intensity.

### Statistical analysis

Fisher’s exact test was used to compare qualitative data and the Wilcoxon signet-rank test (one side) for quantitative data. Correlation between LAT1 score and ratio SUVmax lesion/SUVmax striatum was calculated using non parametric Spearman test. All the tests were considered significant at a 5% type I error rate (p<0.05). Statistical analyses were performed using SPSS version 11.0 (Statistical Package for Social Sciences, SPSS inc; Chicago, USA).

### Positron emission tomography with [18F] FDOPA

Since june 2013, in our institutions (Centre Universitaire de Nice et Centre Antoine Lacassagne), BM patients previously treated by radiotherapy were followed by [18F] FDOPA-PET when IRM failed to discriminate conclusively between tumor recurrence and radionecrosis. Patients were included in the clinical trial IMOTEP (RECF2013) and gave a written consent for the use of tumor sample in research. A 10 min static PET-CT was performed 20 min post injection of 2 MBq/kg of ^18^F-DOPA (mCT-Siemens; OSEM 5 it and 24 subsets). MRI T1 weighted images were co-registered to PET data. Images were graded visually using Lizarraga et al. criteria [12], comparing tumour uptake with striatal activity: 0 lesion not visible, 1 uptake inferior to striatum, 2 lesion uptake equal to striatum and 3 uptake higher than striatum. PET was considered positive for a score > 1. For each case, Standard Uptake Value Maximum (SUVmax) in lesion and in contralateral striatum were measured. A Ratio SUVmax lesion/SUVmax striatum > 0.75 was considered to be evocative of recurrence [3]. Among patients with BM followed by [18F] FDOPA-PET, 11 cases were operated, including 10 cases with [18F] FDOPA uptake suggesting recurrences. One case lacking [18F] FDOPA uptake was evocative of radionecrosis. Two other cases of radionecrosis occurring after irradiation of gliomas (ratio SUVmax lesion/SUVmax striatum ≤0.75), were added. Thus, a total of three cases of radionecrosis were investigated and compared to cases of recurrence. In all three cases, neurosurgical resection was carried out in order to reduce symptoms.

## Results

### Description of LAT1 and CD98 expressions in BM

**Fig 1:**
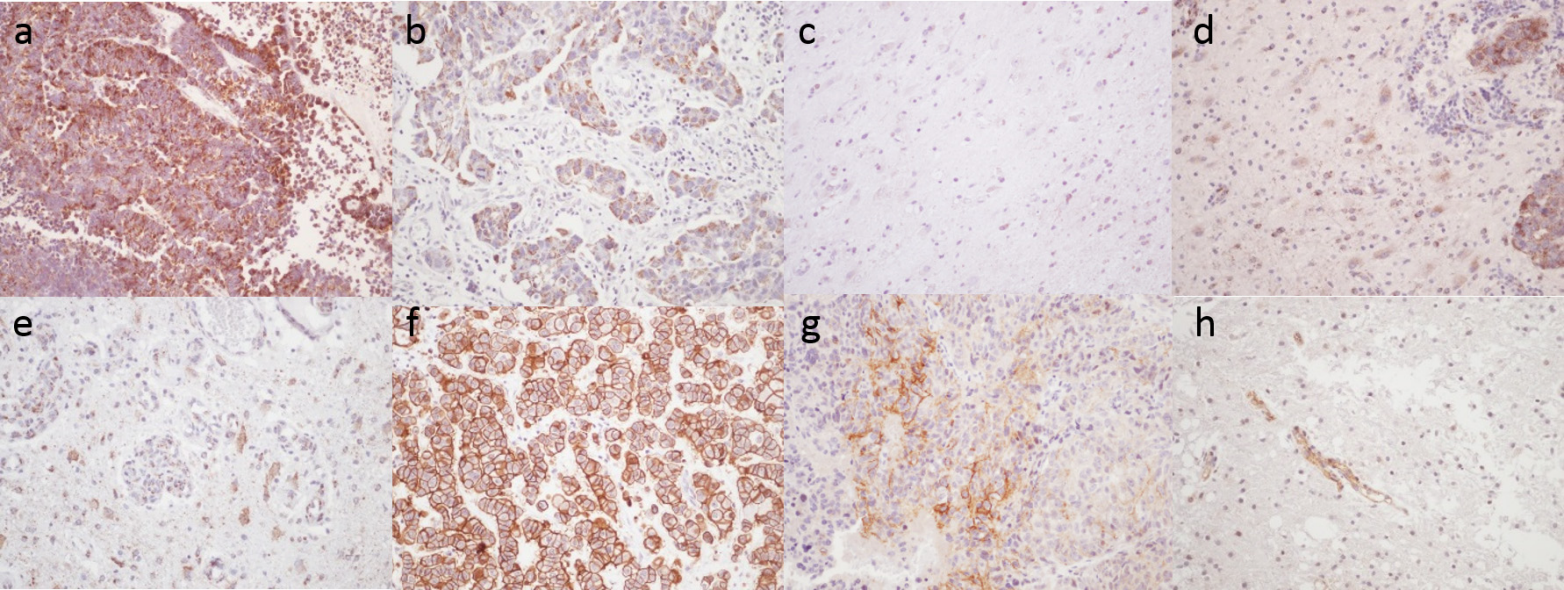
**Expression of LAT1 and CD98 in brain metastases and in non-tumoral brain**: a) strong and diffuse expression of LAT1 in a BM from small cell lung carcinoma, b) heterogeneous staining of LAT1 in a BM of lung squamous cell carcinoma, c) non-tumoral brain negative for LAT1, d) moderate expression of LAT1 in reactive astrocytes at the periphery of a BM, e) strong membrane expression of CD98 in a BM of lung adenocarcinoma, f) focal expression of CD98 in a BM of a lung squamous cell carcinoma, g) CD98 is only expressed in endothelial cells in non-tumoral brain, h) CD98 expression was rarely detected in reactive astrocytes. Immunohistochemistry, x 200.

The level of LAT1 and CD98 expression was assessed by immunohistochemistry in 67 BM. LAT1 expression was detected in 66 out of 67 BM (98.5%) and is detailed in Table 2 and in S1 Table. Its expression was often detected in both the cytoplasm and membrane of tumor cells, less commonly in the cytoplasm alone (Fig 1). The intensity of staining was weak in 16 cases (24.3%), moderate in 28 cases (42.4%) and strong in 22 cases (33.3%). The proportion of LAT1 positive cells varied from 2% to 100% among the cases, with a mean value of 63%. The LAT1 score varied from 0 to 400, with a mean score of 202.

**Table 2:** Expression of LAT1 and CD98 in brain metastases and in non-tumoral brain.

	LAT1			CD98		
	Brain metastases n = 67	Non tumoral brain n = 53	p value	Brain metastases n = 67	Non tumoral brain n = 53	p value
**Positive cases (%)**	66 (98.5%)	34 (64.2%)	p<0.001	40 (59.7%)	5 (9.4%)	p<0.001
**Staining intensity**			p<0.001			p<0.001
weak	16 (24.3%)	20 (58.8%)		25 (62.5%)	3 (60%)	
moderate	28 (42.4%)	13 (38.2%)		11 (27.5%)	2 (40%)	
strong	22 (33.3%)	1 (3%)		4 (10%)	0	
**Rate of positive cells**			p<0.001			p<0.001
range	2–100%	1–90%		1–100%	0–20%	
mean	63%	18.1%		29.5%	0.94%	
**Score**			p<0.001			p<0.001
range	0–400	0–270		0–392	0–60	
mean	202	46.17		79.67	2.35	

CD98 expression was detected in 40 cases of BM (59.7%). Its expression was predominantly located in the membrane of tumor cells, and was rarely cytoplasmic (Fig 1). The intensity of staining was weak in 25 cases (62.5%), moderate in 11 cases (27.5%) and strong in 4 cases (10%). The proportion of CD98 positive cells ranged from 1 to 100%, with a mean value of 29.5%. The CD98 score varied from 0 to 392 with a mean score of 79.67 (Table 2 and S1 Table).

LAT1 and CD98 were co-expressed in 29 cases (43%). There was no correlation between LAT1 and CD98 expressions in terms of staining intensity, rates of positive cells and scores.

There was no difference in LAT1 or CD98 expression either between histological subtypes of BM, or between different origins of primary tumor (lung versus others), in terms of intensity of staining, rate of positive cells or score.

In conclusion, we showed that LAT1 expression was almost universal and CD98 expression frequent in BM.

### Comparison of LAT1 and CD98 expressions between recurrent and newly occurring BM

Tumors showing expression of LAT1 and CD98 have been documented to have a more aggressive behavior. We hypothesized that their expression might be increased in recurrent BM compared to newly occurring BM. Hence, we compared their expression in 18 cases of recurrent BM with 49 cases of newly occurring BM (S1 Table).

LAT1 expression was as frequent in newly occurring BM (48/49 cases) as in recurrent BM (18/18 cases) but recurrent BM contained significantly more positive cells (mean of 78%) and a higher score (mean of 252) than newly occurring BM (mean of 57.5% positive cells, p = 0.003, and a mean score of 184, p = 0.034).

There was no difference in CD98 expression between recurrent and newly occurring BM in terms of frequency (61.1% versus 61.2%), staining intensity (p = 0.45), rate of positive cells (mean of 38.4% versus 26.2%, p = 0.23), and score (mean score of 105 versus 70.8, p = 0.2).

In conclusion, our results showed a higher expression of LAT1 in recurrent BM as compared to newly occurring BM due to an increase in the number of positive cells in the former. CD98 expression remained unchanged.

### Comparison of LAT1 and CD98 expressions between BM and non-tumoral brain tissue

In order to determine whether overexpression of LAT1 and CD98 detected in BM were specific for BM as compared to non-tumoral brain tissue (NT), we studied their expression in 53 samples of NT.

LAT1 expression was detected in 34 of 53 cases of NT (64.2%). Positive cells were identified as astrocytes. The immunostaining was predominantly localized in the cytoplasm and less commonly in the membrane (Fig 1). The intensity of staining in NT, which was predominantly weak to moderate, was significantly weaker than the intensity of staining in the BM (Table 2). The proportion of LAT1 positive cells and the score in NT were significantly lower than for BM (Table 2 and S1 Table).

CD98 expression was detected in 5 of 53 cases of NT (9.4%). Expression of CD98 was detected in the cytoplasm or in the membrane of reactive astrocytes (Fig 1). The staining intensity was weak (3 of 5 cases) or moderate (2 of 5 cases) and was significantly weaker than detected in BM (Table 2). The proportion of CD98 positive cells and the score in NT were significantly lower than for BM (Table 2 and S1 Table).

Our results suggest that strong expression of LAT1 and expression of CD98 are specific for BM as compared to NT.

### Correlation between [18F] FDOPA-PET imaging and LAT1 expression

**Fig 2:**
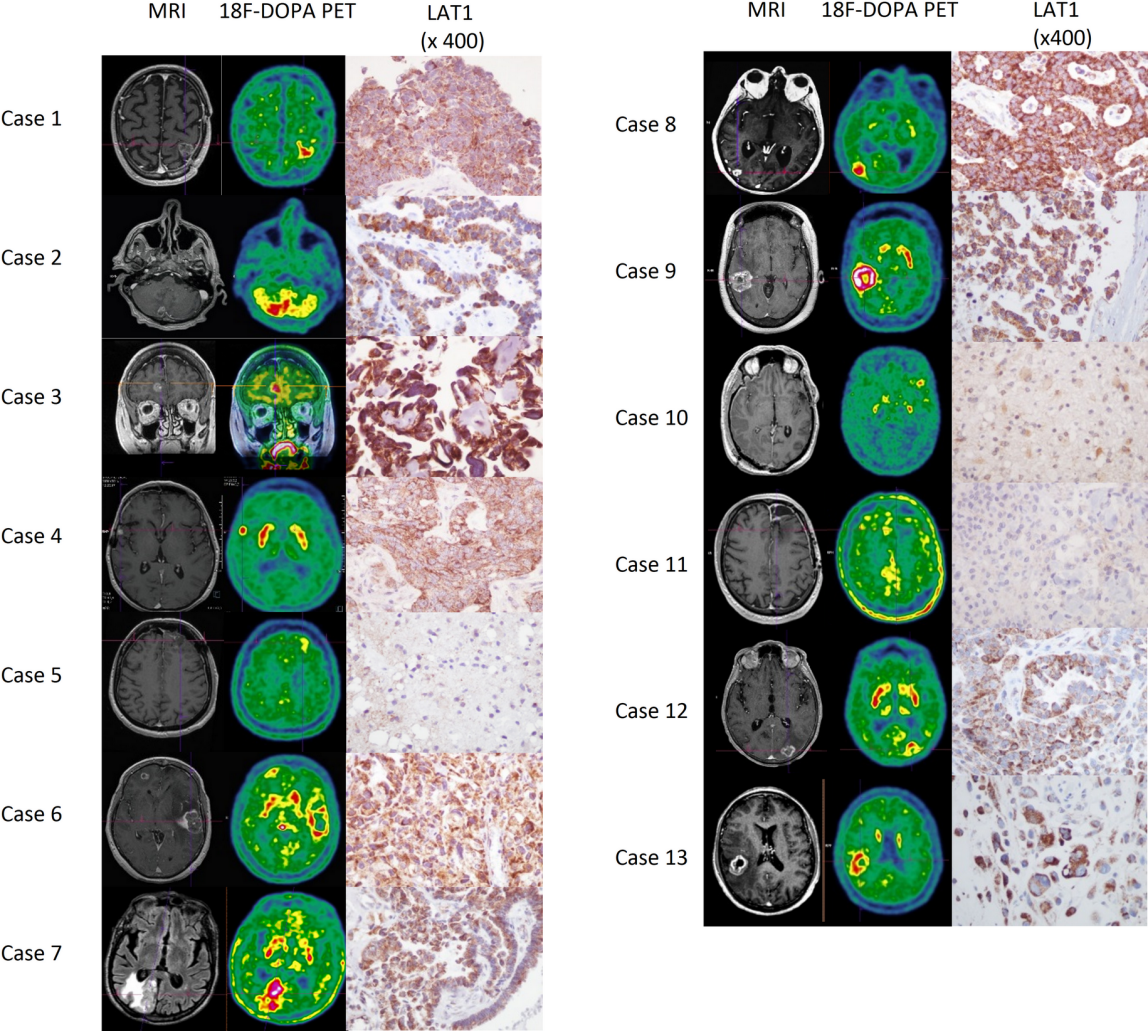
correlation between [18F] FDOPA PET imaging and LAT1 expression in recurrent BM after radiotherapy and in radionecrosis. All the 10 cases which were positive with [18F] FDOPA PET (cases 1 to 4, 6 to 9, 12 and 13), were recurrent BM and displayed LAT1 overexpression with immunohistochemistry. Two cases (cases 5 and 11) corresponding to radionecrosis, lacked [18F] FDOPA uptake and LAT1 expression. One case of radionecrosis (case 10) showed both a low [18F] FDOPA and a low LAT1 expression in reactive astrocytes respectively.

Of the 11 cases of previously irradiated BM investigated both by [18F] FDOPA-PET and histology, 10 cases were recurrent BM and 1 case was radionecrosis. Due to the fact that radionecrosis is usually medically treated, surgical samples of radionecrosis are rare. To strengthen our study, two additional cases of radionecrosis (in a glioblastoma and in an anaplastic oligo-astrocytoma) which had been investigated by [18]-FDOPA were also immunostained for LAT1 expression. All the recurrent BM were positive with [18F] FDOPA-PET and strongly expressed LAT1 (Fig 2) with a score ranging from 110 to 400 (Table 3). The cases of radionecrosis showed no or low [18F] FDOPA-uptake (ratio SUVmax lesion/SUVmax striatum ≤0.75) and LAT1 expression was weak (Fig 2), with a score ranging from 16 to 100 (Table 3). In cases of radionecrosis, LAT1 was expressed by reactive astrocytes around necrosis. Notably, two cases of radionecrosis (cases 5 and 11) displayed no visual uptake and a very low score of LAT1 expression (respectively 16 and 45) and the third case (case 10) showed both a low [18F] FDOPA-uptake (ratio SUVmax lesion/SUVmax striatum = 0.75) and a higher level of LAT1 expression in reactive astrocytes relative to the two previous cases (score 100). In the series, a low LAT1 score (≤ 100) was significantly associated with a low [18F] FDOPA-uptake (ratio SUVmax lesion/SUVmax striatum ≤ 0.75) (p = 0.003) and the intensity of [18F] FDOPA-uptake was correlated with the level of LAT1 expression (p = 0.037).

**Table 3:** Correlation between [18F] FDOPA uptake and LAT1 expression.

Cases	Histology of previous brain tumor	18F-DOPA-PET	SUVmax lesion	Ratio SUVmax lesion/SUVmax striatum	Histology of the new lesion	LAT1 score
**1**	Lung squamous carcinoma	+	5.2	1.026	recurrence	255
**2**	Lung adenocarcinoma	+	5.26	1.069	recurrence	288
**3**	Ovarian adenocarcinoma	+	5.03	0.862	recurrence	400
**4**	Breast adenocarcinoma	+	4	1.108	recurrence	240
**5**	Melanoma	-	2.4	0.57	radionecrosis	16
**6**	Lung adenocarcinoma	+	6.03	0.92	recurrence	240
**7**	Lung adenocarcinoma	+	2.81	1.12	recurrence	294
**8**	Oesophageal adenocarcinoma	+	5.74	1.121	recurrence	400
**9**	Breast adenocarcinoma	+	6.7	1.54	recurrence	160
**10**	Glioblastoma	+/-	4.45	0.75	radionecrosis	100
**11**	Anaplastic oligo-astrocytoma	-	1.76	0.56	radionecrosis	45
**12**	Lung adenocarcinoma	+	2.36	0.78	recurrence	110
**13**	Melanoma	+	5.8	0.98	recurrence	360

These results suggested that AA-PET uptake in recurrent BM might be associated with LAT1 overexpression whereas the absence of uptake in radionecrosis may be due to a low expression of LAT1.

## Discussion

The differential diagnosis between recurrent BM and radionecrosis remains a clinical challenge in the follow-up of patients with BM because tumor recurrence and post-irradiation changes may be similar on MRI. Positron emission tomography using radiolabeled amino acid (PET-AA) appears to be promising in the management of BM, because AA-uptake appears to be specific for tumor cells. The amino acid transporter LAT1 and its cofactor CD98 which are involved in AA uptake in brain lesions [18–20] are overexpressed in numerous tumors but there is no data concerning their expression in BM. Our study showed that LAT1 expression is almost always seen in BM and that CD98 is expressed by almost two thirds of BM, regardless of the histological type and the origin of the tumor. We selected a cohort of 67 BM of various histological subtypes and from different primary sites, in order to define the status of LAT1 and CD98 expression in metastatic tissues reflecting the epidemiology of BM. LAT1 and CD98 expression were independent of the histological subtype and of the primary origin of the tumor, which could be explained by the necessity of tumor cells, regardless of origin, to actively uptake AA for growth and proliferation. Nevertheless, it may be possible that differences do exist between histological subtypes which we could not identify because of the heterogeneity and the small size of our cohort. Such differences between histological subtypes have already been described for primary tumors. For example, it has been shown in lung tumors that LAT1 expression is higher in squamous carcinoma and in large cell carcinoma than in adenocarcinoma [27].

In our study, LAT1 and CD98 expression were studied by immunohistochemistry which enables the correlation of protein expression with cell morphology. LAT1 and CD98 expression were defined on the one hand by the proportion of positive cells and on the other hand by the staining intensity. The score which takes account of the heterogeneity of the staining is more representative of the overall protein expression. Nevertheless, immunohistochemistry does not inform as to whether proteins are functional. CD98 is considered to be necessary for the function of LAT1 hence, we studied the expression of both proteins. Although LAT1 and CD98 expression was very common in BM, we found co-expression in only 43% of cases. Nevertheless, this result does not exclude the possibility that the transporter was functional in a higher proportion of cases. Firstly, the staining threshold (>10%) used in our study to determine positivity, based on data from the literature, has been arbitrarily defined and has never been correlated with a functional activity threshold. Secondly, it has been shown that exclusive expression of LAT1 is sufficient to increase transportation activity, in particular in epithelial cells [30]. Finally, only a comparison between transporter expression and its activity, *in vitro* and *in vivo*, could answer this question about its functional status.

In our study, LAT1 was expressed in nearly all BM and much more frequently than has been previously reported in primary tumors. For example, LAT1 expression in primary lung cancer has ranged from 36.8% to 88.3% [24,31] and has been detected in only 43.4% of breast cancer [25]. This difference could be explained by the use of different antibodies in the studies. It is considered that it is more likely that LAT1 overexpression in BM as compared to primary tumors could reflect the correlation of increased LAT1 expression with tumor aggressiveness and stage of the disease [26–28]. In a study comparing pairs of primary tumors and their corresponding metastases, Kaira *et al*. [28] described stronger expression of LAT1 in metastases. This correlation between LAT1 expression and tumor aggression could possibly clarify why we observed a stronger expression in recurrent BM as compared to newly occurring BM. Furthermore, it has been shown that LAT1 expression was an independent prognostic factor in NSCLC [32], in breast cancer [25], in hepatocellular carcinoma [33], in pancreatic carcinoma [34,35] and in prostatic carcinoma [36]. A few studies also reported that LAT1 expression was correlated with chemoresistance in NSCLC [31] and with radioresistance and chemoresistance in rectal carcinoma [37], but the mechanisms of such resistance are not currently understood. Thus, strong LAT1 overexpression in recurrent BM may indicate a greater aggression and resistance to treatment.

Our data showed that LAT1 overexpression was specific for metastatic tissue as compared to healthy brain. For the first time, we consider that there is a biological basis to explain the difference in PET-AA uptake between BM and normal brain which has been observed in clinical practice. Our results suggest that PET-AA should be able to discriminate recurrent BM from radio-induced lesions. In our study, we found that [18F] FDOPA uptake and LAT1 expression are features of recurrent BM, whereas radionecrosis case lacked both or showed non significant [18F] FDOPA uptake and LAT1 expression. The low uptake of [18F] FDOPA in the case of radionecrosis may be explained by a slight increase of LAT1 expression by reactive astrocytes, as we observed in case 10. In the present study, a lack or low expression of LAT1 was clearly associated with no or low [18F] FDOPA uptake, and seems to be the hallmark of radionecrosis. Due to LAT1 known biological function, these results suggest that LAT1 most likely contributes to AA-uptake. Furthermore, we found that intensity of [18F] FDOPA uptake was statistically correlated with the score of LAT1 expression, which strengthen the hypothesis that LAT1 is crucial for [18F] FDOPA uptake. Nevertheless, some cases in our study displayed strong [18F] FDOPA uptake and moderate LAT1 expression and inversely. Such discordance between intensity of [18F] FDOPA uptake and level of LAT1 expression in our study may be due to sampling problems (for example if LAT1 expression is not assessed in the part of the lesion corresponding to the SUV max). The existence of other mechanisms than LAT1 involved in the AA uptake may also be discussed. Indeed, although [18F] FDOPA uptake has been shown to be dependent on LAT1, the mechanisms involved are not completely elucidated. Thus, LAT1 appears to be relevant for [18F] FDOPA uptake but a linear correlation between [18F] FDOPA uptake and LAT1 expression remains to be clearly demonstrated by further studies. Our series was limited by a small number of cases investigated both by [18F] FDOPA-PET and histology and investigations of larger cohorts are required to draw conclusions. The main limitation of such studies in humans is the rarity of cases of resected radionecrosis. In our practice, only previously irradiated BM cases for which MRI differential diagnosis between recurrence and radionecrosis was challenging, were investigated by [18F] FDOPA-PET (since 2013). Among them, only a minority were operated and very rare cases corresponded to radionecrosis. In our study, the cases of radionecrosis were resected to reduce symptoms. In the study of Lizarraga *et al*.[12], among 83 BM investigated by [18F] FDOPA-PET, only 9 were validated by histological examination including 2 cases of radionecrosis. In practice, cases of radionecrosis when identified are often medically treated and resection is more rarely indicated. Thus, both histological and PET data are rarely available in cases of radionecrosis, limiting the possibilities to compare both LAT1 expression and AA-PET data between recurrent BM and radionecrosis in humans.

The main limitation of [18]FDG PET for brain tumor evaluation is the lack of contrast between normal brain and lesion, relative to high physiological glucose uptake in normal brain. In PET-AA, the contrast between normal brain and brain tumor is clear and is based on the differential capacities of the normal and pathological tissues for AA uptake. In the present study, we demonstrated a clear difference of LAT1 expression between normal/irradiated brain and metastatic tissue. These results enable us to understand better the specificity of PET-AA for the evaluation of BM as compared to [18F]FDG PET. Nevertheless, we observed that radionecrosis may display a slight increase of AA uptake also, suggesting that all the mechanisms involved in AA uptake in irradiated brain are not well elucidated. Consequently, at this time, the eventuality of “non specific” PET-AA uptake in radionecrosis can not be firmly excluded.

In conclusion, LAT1 overexpression was specific for BM as compared to healthy brain and could explain the specific AA uptake by BM on PET-AA usually observed in clinical practice. This suggests that LAT1 plays a crucial role in AA uptake in BM. Nevertheless, further studies are required to better comprehend all the mechanisms involved in the AA uptake in the diseased brain.

## Supporting Information

S1 Table Expression of LAT1 and CD98 in BM and NT. (XLS)
